# The influence of P-glycoprotein expression and its inhibitors on the distribution of doxorubicin in breast tumors

**DOI:** 10.1186/1471-2407-9-356

**Published:** 2009-10-06

**Authors:** Krupa J Patel, Ian F Tannock

**Affiliations:** 1 Department of Medical Biophysics, University of Toronto, Toronto, ON, Canada; 2 Medical Oncology and Hematology, Princess Margaret Hospital and University of Toronto, Toronto, ON, Canada

## Abstract

**Background:**

Anti-cancer drugs access solid tumors via blood vessels, and must penetrate tumor tissue to reach all cancer cells. Previous studies have demonstrated steep gradients of decreasing doxorubicin fluorescence with increasing distance from blood vessels, such that many tumor cells are not exposed to drug. Studies using multilayered cell cultures show that increased P-glycoprotein (PgP) is associated with better penetration of doxorubicin, while PgP inhibitors decrease drug penetration in tumor tissue. Here we evaluate the effect of PgP expression on doxorubicin distribution *in vivo*.

**Methods:**

Mice bearing tumor sublines with either high or low expression of PgP were treated with doxorubicin, with or without pre-treatment with the PgP inhibitors verapamil or PSC 833. The distribution of doxorubicin in relation to tumor blood vessels was quantified using immunofluorescence.

**Results:**

Our results indicate greater uptake of doxorubicin by cells near blood vessels in wild type as compared to PgP-overexpressing tumors, and pre-treatment with verapamil or PSC 833 increased uptake in PgP-overexpressing tumors. However, there were steeper gradients of decreasing doxorubicin fluorescence in wild-type tumors compared to PgP overexpressing tumors, and treatment of PgP overexpressing tumors with PgP inhibitors led to steeper gradients and greater heterogeneity in the distribution of doxorubicin.

**Conclusion:**

PgP inhibitors increase uptake of doxorubicin in cells close to blood vessels, have little effect on drug uptake into cells at intermediate distances, and might have a paradoxical effect to decrease doxorubicin uptake into distal cells. This effect probably contributes to the limited success of PgP inhibitors in clinical trials.

## Background

Effective systemic treatment of solid tumors requires that constituent cells be sensitive to the drug(s) used and that the drug(s) be able to achieve a concentration within the cells sufficient to cause cytotoxicity. The ability of anti-cancer drugs to gain access to all of the viable cells in solid tumors depends on efficient delivery of drugs through the vascular system, and on penetration of drugs to tumor cells that are distant from blood vessels. Solid tumors have an inefficient bloody supply with tortuous and leaky vessels, large intercapillary distances and intermittent blood flow. There is evidence that several anticancer drugs have poor penetration of tumor tissue from blood vessels [1-5]. In particular there are steep gradients of decreasing doxorubicin fluorescence with increasing distance from blood vessels in tumors grown in mice and in human breast cancer, suggesting that limited drug penetration may be an important cause of resistance to treatment [6,7].

Many of the anticancer drugs in clinical use are natural products, or derivatives of natural products (e.g. anthracyclines such as doxorubicin, vinca alkaloids such as vincristine and taxanes) and they share common mechanisms of resistance. Many of these drugs are high affinity substrates for energy-dependent membrane transporter proteins that act to pump drugs out of cells. The best characterized of these drug efflux pumps is P-glycoprotein (PgP) [8-10]. P-glycoprotein is widely expressed in many human cancers including those of the liver, pancreas, kidney, ovary and breast. PgP is encoded by the multidrug resistance (MDR1) gene in humans and is a member of a large family of ATP-dependent transporters known as the ATP-binding cassette (ABC) family. Levels of PgP correlate with drug resistance in several different cancers [11,12]. This has led to the development of agents that reverse resistance to PgP substrates by inhibiting the action of the pump. However, while phase II studies have suggested that PgP inhibitors might increase drug sensitivity [13,14], the majority of randomized controlled trials evaluating PgP substrate drugs used in combination with or without PgP modulators, have not shown significant improvements in outcome [15]. Various factors have been attributed to the failure of PgP modulators in clinical trials such as high levels of toxicity and pharmacokinetic interactions with the anticancer drugs [15,16]. In the last decade novel techniques using liposome-encapsulated doxorubicin in combination with PgP inhibitors such as verapamil and PSC 833 have been developed to increase intracellular drug concentration and minimize toxicity [17-19], however clinical data showing significant improvements in outcome have yet to be reported. Thus, other factors may also contribute in explaining the limited effectiveness of PgP inhibitors *in vivo*.

Studies have revealed differences in drug distribution within *ex vivo *tumor tissue models containing cells expressing low levels of PgP and those with high PgP expression, and these distributions can be modified by PgP inhibitors [20,21]. The role of PgP and its inhibitors to affect drug distribution within the microenvironment of solid tumors has not been studied. In tumors that express low levels of PgP, it has been demonstrated that large quantities of drug remain in the cell layers closest to blood vessels, while distal cells acquire little to no drug [7,22]. We hypothesized that PgP, by preventing cellular accumulation of drug in cells close to blood vessels would allow greater quantities of drug to be available to distal cells. Studies of drug penetration through multilayered cell cultures (MCC) support this hypothesis [20]. Although cellular accumulation of drug is important for cytotoxicity, if it is localized to perivascular regions, then there is likely to be a limited overall therapeutic effect on the tumor. Here we examine in solid tumor models the trade-off between drug uptake and drug distribution that is presented by PgP overexpression and PgP inhibitors.

## Methods

### Drugs and reagents

Doxorubicin (Pharmacia, Mississauga, Canada) was purchased from the hospital pharmacy as a solution at a concentration of 2 mg/mL. Purified rat anti-mouse CD31 (platelet/endothelial adhesion molecule 1) monoclonal antibody was purchased from BD PharmMingen (Mississauga, Canada), and Cy3-conjugated goat anti-rat IgG secondary antibody was purchased from Jackson ImmunoResearch Laboratories, Inc. (Pennsylvania, USA). The first-generation PgP inhibitor, verapamil was purchased from Sigma Laboratories (Oakville, Canada). The second-generation inhibitor, PSC 833 or valspodar, was generously provided by Novartis (Basel, Switzerland).

### Tumor models

The mouse mammary sarcoma EMT6, and its PgP-upregulated variant AR1, were provided originally by Dr Peter Twentyman, Cambridge UK. The human breast cancer cell line MCF-7 was obtained from the American Type Culture Collection (ATCC; Virginia, USA), and its PgP upregulated variants BC19 and MCF-ADR were provided by Dr. Marilyn Morris (Buffalo, NY), and Dr. Kenneth Cowan (Nebraska).

EMT6 and MCF-7 cells were maintained as monolayers in α-MEM and RPMI media respectively, supplemented with 10% FCS at 37°C in a humidified atmosphere of 95% air plus 5% CO_2_. The AR1, BC19 and MCF-7/ADR cell lines were maintained in similar conditions to the parental lines with the exception that the media contained 10 μg/mL doxorubicin.

Tumors were generated by subcutaneous injection of 1 - 5 × 10^6 ^exponentially growing cells into the left and right flank regions of 6-8 week old Balb/C (EMT6 and AR1) or athymic nude (MCF-7, BC19 and MCF-7/ADR) female mice. Mice were housed five per cage in our animal colony. Sterile water and food were given ad libitum. All procedures were carried out following approval of the Institutional Animal Care Committee.

#### Evaluation of Doxorubicin Distribution

Tumor-bearing mice were divided randomly into groups of five and were treated when the mean tumor diameter was in the range of 8-12 mm. Animals were treated with doxorubicin, doxorubicin and a PgP inhibitor, or phosphate-buffered saline (CaCl_2_-MgCl_2_). Doxorubicin was given intravenously at a dose of 25 mg/kg to facilitate detection and quantification of drug auto-fluorescence. Each PgP inhibitor was administered intraperitoneally two hours prior to doxorubicin treatment at a dose of 25 mg/kg. Animals were killed 10 minutes after doxorubicin injection and the tumors were excised. The tissues were embedded immediately in OCT compound, frozen in liquid nitrogen, and stored at -70°C prior to tissue sectioning and immunohistochemical staining. Cryostat sections 10 μm thick were cut at 3 levels approximately 100 μm apart from each tumor, mounted on glass slides and allowed to air dry.

#### Fluorescence Imaging

Doxorubicin auto-fluorescence was detected utilizing an Olympus Upright BX50 microscope with a 100 W HBO mercury light source equipped with 530 to 560 nm excitation and 573 to 647 nm emission filter sets. Tissue sections were imaged with a Photometrics CoolSNAP HQ2 (monochrome for fluorescence imaging) camera and tiled using a motorized stage so that the distribution of doxorubicin was obtained for the entire tissue section. All images were captured in 8-bit signal depth and subsequently pseudo-colored.

Blood vessels in tissue sections were recognized by the expression of CD31 on endothelial cells. Subsequent to imaging of doxorubicin, tissue sections were stained with a rat anti-CD31 antibody (1/100) followed by a Cy3-conjugated goat anti-rat IgG secondary antibody (1/400). Tissue sections were re-imaged in an identical way to that used to capture doxorubicin fluorescence.

#### Image analysis

Composite images of doxorubicin and CD31 were generated utilizing Media Cybernetics Image Pro PLUS software (version 5.0). Images displaying anti-CD31 staining were converted to a black and white binary image, and small white objects were removed as artifacts based on conservative estimation of minimal capillary diameter [23]. The resultant image was overlaid with the corresponding field of view displaying doxorubicin fluorescence resulting in an 8 bit black and white image with blood vessels identified by pixels with intensities between 250-255 (white) and doxorubicin ranging from 0-249. Several regions, 1.6 mm^2 ^in area, with moderate blood vessel density were selected from each tissue section. Blood vessel density was determined by quantifying the number of pixels with intensities between 250-255 as a percent of the number of pixels with intensities from 1-249 using Image J software. Areas of necrosis and staining artifact were excluded. To minimize noise from tissue auto-fluorescence a minimum signal level just below threshold for detection of doxorubicin was set for each tissue section; this was based on an average background reading from regions without nuclear fluorescence/staining. The pixel intensity (the area of each pixel was 0.4 μm^2^) and distance to the nearest vessel for all pixels within the selected region of interest above threshold were measured with a customized algorithm.

Doxorubicin intensity (I) was averaged over all pixels at a given distance (*x*) from the nearest vessel and plotted as a function of distance to the nearest vessel. Linear regression was performed to correlate the average doxorubicin fluorescence intensity with distance from the nearest blood vessel; the slope of the linear regression was statistically compared between treatment groups using ANOVA and subsequent t-tests. All linear regressions were statistically significant and residual plots showed no consistent patterns.

A model comparing doxorubicin distribution in PgP overexpressing tumors pre-treated with either saline or a PgP inhibitor was generated using the mean slope and y-intercept from the linear regressions for both the murine and xenograft tumor types.

#### Growth delay studies

Mice bearing EMT6 or AR1 tumors were divided into six groups of 4-5 mice each and treated with either saline, doxorubicin alone (8 mg/kg i.v., verapamil alone (25 mg/kg i.p.), PSC 833 alone (25 mg/kg), verapamil + doxorubicin (25 mg/kg + 8 mg/kg) or PSC 833 + doxorubicin (25 mg/kg + 8 mg/kg). In the latter two groups mice were treated with the PgP inhibitor 2 hours prior to doxorubicin treatment. Every 2-3 days the length and width of the tumors were measured using calipers and tumor volume was calculated. Measurements were taken until tumors reached their maximum limit in size. The body weight of the mice was also measured.

## Results

### Blood vessel density

Blood vessel density within areas of interest was measured and found to be within a small range. Murine tumors are significantly more vascular than the xenografts (Table 1). Within a given tumor section there are small areas with high blood vessel density where no gradients of decreasing doxorubicin fluorescence are observed, but the majority of the tumor section is composed of areas of moderate blood vessel density with gradients of decreasing doxorubicin fluorescence with increasing distance from vessels.

**Table 1:** Characteristics of wild-type (EMT6, MCF-7) and PgP overexpressing tumors (AR1, BC19) at 10 minutes after treatment with doxorubicin (DOX) or with pre-treatment 2 hours earlier with inhibitors of PgP.

Tumor Type	PgP vs. Wild-type	Treatment	Blood Vessel Density	DOX uptake at distance from the nearest blood vessel			Gradient of Decreasing DOX Intensity
					
				10-20 μm	50-60 μm	110-120 μm	
EMT6	Wild Type	DOX	4.7 ± 1.2	29.4 ± 6.1^*a*^	15.6 ± 1.9	11.3 ± 2.4	-0.23 ± 0.08^*c*^
AR1	Overexpress PgP	DOX	6.0 ± 1.4	17.1 ± 2.9^*ab*^	10.5 ± 2.8^*c*^	7.4 ± 1.6	-0.11 ± 0.03^*cde*^
AR1	Overexpress PgP	Verapamil + DOX	4.4 ± 1.1	24.7 ± 8.3	13.7 ± 4.2	9.2 ± 4.0	-0.18 ± 0.06^*d*^
AR1	Overexpress PgP	PSC 833 + DOX	4.7 ± 1.3	34.0 ± 4.3*^b^*	16.4 ± 2.3^*c*^	12.1 ± 3.7	-0.27 ± 0.04*^e^*

MCF-7	Wild Type	DOX	2.6 ± 0.7	23.8 ± 4.2*^f^*	13.7 ± 2.5	10.4 ± 2.3	-0.16 ± 0.05*^h^*
BC19	Overexpress PgP	DOX	2.7 ± 0.4	13.0 ± 4.3*^fg^*	9.6 ± 2.8	8.7 ± 3.5	-0.05 ± 0.03*^hij^*
BC19	Overexpress PgP	Verapamil + DOX	3.6 ± 0.8	22.1 ± 5.9	13.9 ± 3.7	9.2 ± 1.1	-0.14 ± 0.04*^i^*
BC19	Overexpress PgP	PSC 833 + DOX	2.9 ± 0.7	24.2 ± 4.1*^g^*	14.4 ± 2.5	10.9 ± 3.6	-0.17 ± 0.05*^j^*

Top and bottom panels were tested separately for significant differences using ANOVA and subsequent t-tests

Within the top panel, *^a-e^* represent the pairs of data that are statistically significant (p < 0.05)

Within the bottom panel, *^f-j^* represent the pairs of data that are statistically significant (p < 0.05)

### PgP overexpression and doxorubicin distribution

**Figure 1:**
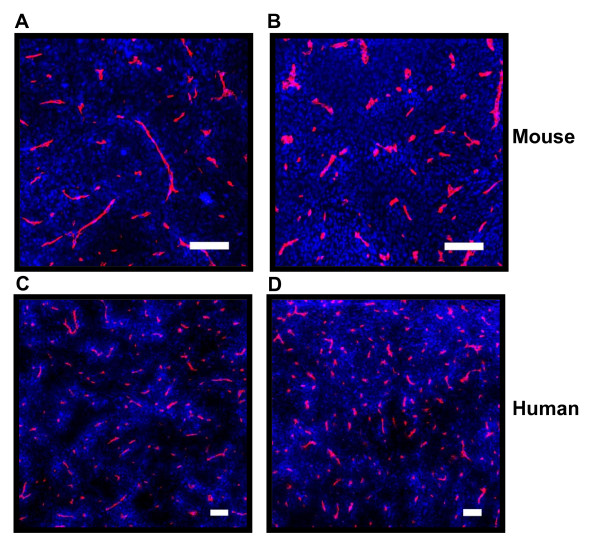
**Distribution of doxorubicin in solid tumors**. Murine tumors EMT6 (A) and its PgP overexpressing subline AR1 (B) and MCF-7 human breast cancer xenograft (C) and its PgP overexpressing subline BC19 (D) were resected from Balb/C and nude mice, respectively. Doxorubicin is shown in blue and blood vessels are shown in red. Note more uniform distribution of doxorubicin in the PgP overexpressing tumors. (Scale bars = 100 μm)

**Figure 2:**
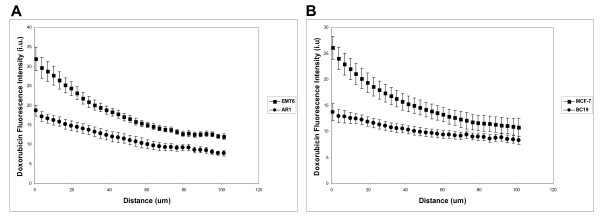
**The gradient of doxorubicin fluorescence intensity in relation to distance from the nearest blood vessel**. Mice-bearing either EMT6 or AR1 tumors (A) (n = 6 tumors each) or MCF-7 or BC19 xenografts (B) (n = 11 and 5 tumors, respectively) were treated with doxorubicin and their tumors were resected, sectioned and imaged. Image analysis was undertaken using customized algorithms. Values represent mean ± standard error.

Representative composite images showing the distribution of doxorubicin 10 minutes after administration in relation to blood vessels of wild-type EMT6 tumors, and in tumors derived from cells that over-express PgP are shown in Figures 1A and 1B; similar images for MCF7/BC19 xenografts are shown in Figures 1C and 1D. A summary of data obtained from these sections is provided in Table 1. In PgP overexpressing tumors, a more homogeneous distribution of doxorubicin is observed as compared to wild-type tumors of both murine and human origin (Figure 1). The gradient of decreasing doxorubicin fluorescence intensity is significantly greater in wild-type tumors that have low levels of PgP expression (Table 1). Whereas wild-type tumors show an exponential decrease in doxorubicin fluorescence with distance from blood vessels, PgP overexpressing tumors show a more linear decrease (Figure 2). Close to blood vessels (i.e. in the first 10 μm), doxorubicin uptake is significantly lower in tumors that overexpress PgP, but at 50-60 μm from blood vessels, the difference in doxorubicin uptake is less and by 110-120 μm, there is no significant difference (Table 1 and Figure 2).

### PgP inhibitors and doxorubicin penetration

**Figure 3:**
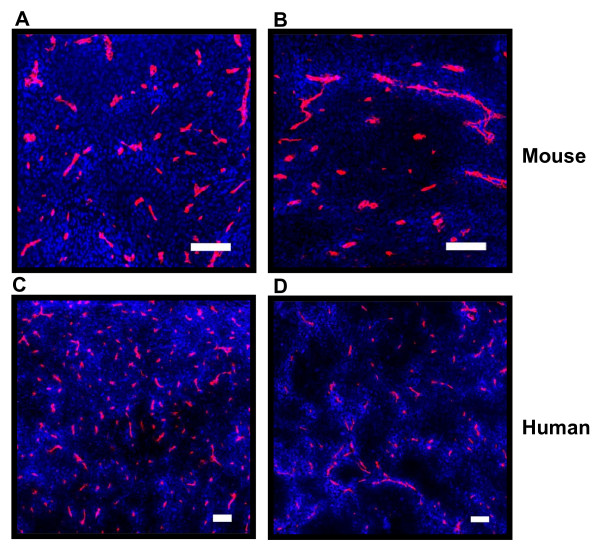
**Distribution of doxorubicin in solid tumors**. Murine AR1 tumors were treated with either doxorubicin (A) or PSC 833 and doxorubicin (C). Similarly, BC19 xenografts were treated with either doxorubicin (B) or PSC 833 and doxorubicin (D). Doxorubicin is shown in blue and blood vessels are shown in red. (Scale bars = 100 μm)

**Figure 4:**
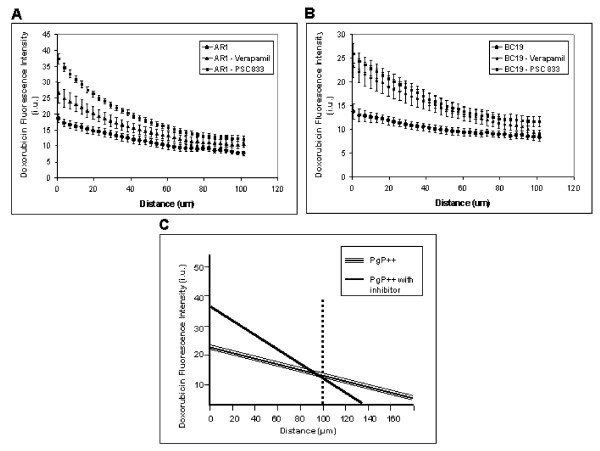
**The gradient of doxorubicin fluorescence intensity in relation to distance from the nearest blood vessel and a model of doxorubicin distribution in solid tumours**. Mice-bearing AR1 tumors (A) or BC19 xenografts (B) were treated with either doxorubicin alone, or pretreated with verapamil or PSC 833 and doxorubicin. Tumors were resected, sectioned and imaged. Image analysis was undertaken using customized algorithms. Values represent mean ± standard error. In panel A, 6, 10 and 9 tumors were analyzed, respectively. In panel B, 6, 7 and 6 tumors were analyzed, respectively. (C) represents a model of doxorubicin distribution without fluouresence interference from neighboring out-of-section blood vessels.

The effects of verapamil and PSC 833 on distribution of doxorubicin in PgP-overexpressing AR1 tumors are shown in Figures 3A and 3B, and for corresponding BC19 xenografts in Figures 3C and 3D. Both PgP inhibitors lead to an increase in uptake of doxorubicin by cells close to blood vessels, but increase the gradient of decreasing intensity so that the distribution is more heterogeneous and similar to that of wild-type tumors (Table 1). Figure 4 shows the distribution of doxorubicin in PgP overexpressing tumors with or without pretreatment with PgP inhibitors. Doxorubicin fluorescence intensity in the first 10 μm from blood vessels is significantly greater in PgP overexpressing tumors that were pretreated with verapamil and PSC 833 in the murine tumor model, and with PSC 833 in the xenograft model. At further distances (110-120 μm), no significant difference is observed in doxorubicin uptake between control tumors and tumors pretreated with PgP inhibitors (Table 1 and Figure 4).

At distances greater than about 90 - 100 μm from blood vessels estimates of doxorubicin fluorescence are subject to noise, most likely due to the influence of closer blood vessels out of the plane of the sections. While in some areas of the tumors, neighboring vessels may contribute doxorubicin fluorescence in distal cells, in other areas this may not be the case and there may be a sharp decrease in doxorubicin. We have shown the average doxorubicin distribution taken from several various areas of interest. Modeling from this data by removing noise from neighboring vessels suggests that the use of potent PgP inhibitors, such as PSC 833 might lead to a paradoxical decrease in the uptake of doxorubicin in tumor cells situated more distant from tumor blood vessels (Figure 4c). According to the model, at around 90-100 μm in the murine model and 100-115 μm in the xenograft model doxorubicin fluorescence intensity is greater in the PgP overexpressing tumors that are treated with doxorubicin alone compared to those pretreated with a PgP inhibitor.

### Growth delay and toxicity

**Figure 5:**
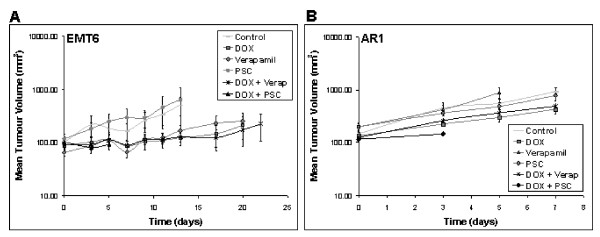
**Tumor growth delay and toxicity studies**. Mice-bearing EMT6 tumors (A) or AR1 tumors (B) were treated with either saline, doxorubicin alone, verapamil alone, PSC 833 alone or pretreated with verapamil or PSC 833 in combination with doxorubicin. Tumor volume and body weight was measured every 2-3 days. Values represent mean ± standard error (n = 5).

Doxorubicin led to modest delay in growth of wild-type EMT6 tumors (Figure 5A) but had no significant effect on PgP overexpressing AR1 tumours. The PgP inhibitors did not modify growth delay for either type of tumor. Body weight was used as an indication of toxicity. Most treatments were well tolerated and caused no significant loss of body weight after treatment. The combination of doxorubicin and PSC 833 was toxic to mice and caused a 20-25% reduction in body weight within the first 3-5 days (data not shown). Mice in this group were killed after 5 days because of this toxicity.

## Discussion

In order for chemotherapy to have an optimal effect against solid tumors, there must be adequate distribution of drugs, such that there is accumulation of drug within all cancer cell populations that can regenerate the tumor. Repopulation of surviving tumor cells between courses of chemotherapy is an important mechanism of drug resistance [24-28], and viable cells distal from blood vessels that do not receive cytotoxic concentrations of drug might be an important source of such repopulation [29].

Drug distribution may be influenced in tumor tissue by the presence of high interstitial fluid pressure, an extensive extracellular matrix, cell-cell contact and the expression of efflux pumps such as PgP on the cell surface. It has been proposed that since PgP and other drug transporters reduce cellular accumulation of their substrates, they might assist in transporting the drugs to neighboring cells farther away from blood vessels, resulting in a net increase in tissue penetration of chemotherapeutic agents [2]. This hypothesis has been supported by the observation of increased penetration of [^14^C] doxorubicin through drug-resistant MCCs (PgP overexpressing) compared to drug-sensitive MCCs (wild-type) [20]; inhibitors of PgP stimulated cellular accumulation of drugs, but resulted in reduced penetration through tumor tissue in this model [20]. While using MCCs provides insight into the penetration of drugs through solid tissue, they lack the 3-dimensional heterogeneity of solid tumors. In studies done using multicellular spheroids, verapamil showed limited reversal of resistance by doxorubicin likely due to its small diameter (approx. 100 μm) and acidic microenvironmental affects on verapamil activity [30,31]. When a wide array of PgP reversal agents including cyclosporin A, verapamil, quinidine, and sodium orthovanadate were used in larger spheroids an increase in doxorubicin fluorescence as far as 80 μm was observed. However, doxorubicin retention at these depths was correlated with an increase in PgP expression, therefore changes in doxorubicin distribution patterns throughout the spheroid tissue due to PgP overexpression and inhibitors could not be assessed [32]. Multicellular spheroids allow good insight into drug fluorescence however, they lack vasculature and certain microenvironmental conditions that lead to specific phenotypes such as multidrug resistance and tumor acidity.

Here, we have investigated the effects of PgP overexpression and PgP inhibitors on doxorubicin distribution in solid tumors grown in mice. The distribution of doxorubicin is quite variable within tumor tissue. In well-vascularized areas drug distribution is relatively uniform; however in most areas of the tumor where there are few blood vessels, distribution to cells distal from blood vessels is limited so that many viable tumor cells have minimal exposure to drug, allowing them to survive and proliferate. For this reason, we selected areas of interest within a tumor section that had low vascular density. We examined doxorubicin distribution 10 minutes after intravenous administration because previous studies in our laboratory have shown no difference in doxorubicin distribution in relation to the nearest blood vessel beyond this time point [7].

Similar to data for MCCs, our results show an increase in doxorubicin distribution in the PgP overexpressing tumors AR1 and MCF-7/ADR compared to their wild-type variants EMT6 and MCF-7, respectively. There is rapid cellular uptake of doxorubicin in wild-type cells and this is largely due to its ability to bind tightly to DNA [33], and in tumors derived from them, large amounts of drug accumulate in cells closest to blood vessels. Doxorubicin is a PgP substrate, that is efficiently pumped out of tumor cells where PgP is highly expressed [16]. Thus, in PgP overexpressing tumors there is less uptake and binding into proximal cells, resulting in a shallower gradient of decreasing doxorubicin fluorescence with increasing distance from tumor blood vessels. These results do not imply that PgP overexpressing tumors allow for more effective drug treatment, since cellular accumulation of drug is essential for cytotoxicity, and PgP expression will decrease relative uptake of substrate drugs in all tumor cells. However, our results demonstrate that PgP efflux can significantly alter the biodistribution of chemotherapeutic agents, such that decreased proximal uptake of drug may be counter-balanced by greater amounts of doxorubicin available to distal cells in solid tumors.

Methods of inhibiting PgP have been studied extensively for over two decades. Many agents that modulate PgP transport such as verapamil and cyclosporin were identified in the 1980s, and were evaluated as chemosensitizing agents. These agents produced disappointing results in clinical trials in part because their low binding affinities necessitated the use of high doses, resulting in unacceptable toxicities [15]. In our studies, a relatively low dose of verapamil (25 mg/kg) was used to determine its effect on drug distribution. While toxicity is likely to be the primary cause of the ineffectiveness of verapamil to improve the sensitivity of tumors to substrate drugs, our studies show that modification of doxorubicin distribution in tumors might be an additional cause of their failure to improve tumor response to chemotherapy.

Second generation PgP modulators include valspodar (PSC 833), biricodar, and dexniguldipine. These agents are more potent and specific than first generation inhibitors [16]. Valspodar has been studied in clinical trials in combination with cytotoxic agents [14,34]. A study by Coley et al [35], that used fresh tumor material from patients with soft-tissue sarcomas indicated that valspodar at 1 nM had a modest effect to increase anthracycline accumulation (by ~20%) in PgP positive samples. In a study of women with epithelial ovarian cancer, the effect was of a similar magnitude; these limited effects might explain in part the disappointing results of clinical trials. Some second generation PgP inhibitors are also, like cytotoxic agents, substrates of cytochrome P450 3A4, an important enzyme involved in metabolism, and the competition between cytotoxic drugs and PgP inhibitors for cytochrome P450 activity has resulted in unpredictable pharmacokinetic interactions. Here we found substantial toxicity when a low dose of PSC 833 was combined with doxorubicin. Marked pharmacologic interactions have been observed in patients treated with this combination, leading to substantial increases in hematologic toxicity [34], and requiring use of lower doses of anticancer drugs when used in combination.

Although cytotoxicity and pharmacokinetic interactions explain partially why PgP inhibitors have not been very effective in clinical trials, a clear explanation for the minimal effects to increase mean cellular accumulation of drug has yet to be provided. Studies to determine cellular accumulation often involve homogenizing tumor tissue and conducting high-performance liquid chromatography (HPLC) [36,37]. This method is limiting, because it does not give an indication of the distribution of drug.

Our study uses a non-orthotopic tumor mouse model to show that tumor cells in areas far from blood vessels where PgP inhibitors have no effect (or possibly even a negative effect) due to changes in distribution of doxorubicin, might counterbalance areas close to blood vessels where uptake of doxorubicin into tumor cells is increased, thus limiting the effectiveness of these inhibitors. While tumor dynamics, vasculature and heterogeneity affect drug distribution, this non-orthotopic mouse model is still limited in fully representing the conditions in human patients.

Computer- simulated mathematical models have shown that adequate drug distribution is a crucial factor in determining drug effectiveness [38]. One model has demonstrated that drug efflux from cells, enhanced by PgP, will result in a longer diffusion length [39]. These mathematical models are powerful tools that can provide important insights about drug distribution, because they can take into account drug pharmacodynamics [40], the spatio-temporal accumulation of drug [41], the link between multiscale approaches [42,43], and the effects of local drug, oxygen and nutrient gradients on tumor growth and response [44]. Future studies integrating our data with computer-simulated mathematical models may be a powerful tool in determining drug distribution and its association with drug effectiveness in human patients.

## Conclusion

We have shown that PgP expression and inhibitors of PgP function can influence doxorubicin distribution in some solid tumors. The distribution of doxorubicin is more heterogeneous in wild-type tumors, than in those where constituent cells express PgP: drug uptake is higher in cells close to blood vessels in wild-type tumors, but there are minimal differences in drug uptake by more distal cells. Both the first-generation inhibitor verapamil and the second-generation inhibitor valspodar alter drug penetration in both a murine tumor and a human xenograft, leading to improved uptake of doxorubicin only in tumor cells within a restricted radius around functional blood vessels. Whether PgP overexpressing tumors are treated with a PgP inhibitor or not, cells distal to blood vessels at approximately 50-120 μm, show no significant difference in doxorubicin fluorescence (Table 1). Our modeling suggests the possibility of a paradoxical effect of PgP inhibitors to cause PgP overexpressing tumors to have increased uptake of doxorubicin in proximal cells, minimal or no effect on drug uptake at intermediate distances from blood vessels but *decreased *drug uptake in more distal cells (Figure 4c). These results emphasize that while limited cellular accumulation of a drug is an important mechanism of drug resistance, if there is a trade-off between uptake into proximal cells and penetration to distal cells, therapeutic effectiveness may be limited, especially for drugs like doxorubicin that have a short half-life in the circulation. Repopulation of tumor cells has been shown to occur in regions far from blood vessels [29], and it is important to consider factors that affect the distribution of chemotherapeutic agents in solid tumors. Numerous clinical trials evaluating the effects of verapamil or valspodar in combination with chemotherapeutic agents such as doxorubicin, vincristine, dexamethasone, cyclophosphamide, paclitaxel have shown that patients given concurrent administration of PgP inhibitors with chemotherapeutic agents have an increased toxicity and show modest or no increase in survival [45-49]. These results provide insight into an important limitation of PgP inhibitors and this principle is likely to be applicable to other membrane-based drug efflux proteins such as multiple drug resistance protein-1 (MRP-1) and suggest the importance of considering drug distribution in the design and development of novel treatment strategies.

## Competing interests

The authors declare that they have no competing interests.

## Authors' contributions

KJP participated in designing, planning and carrying out *in vivo *drug distribution studies, imaging, analysis and growth delay studies as well as drafting and revising the manuscript. IFT conceived the concepts underlying the study, designed the experiments, revised the manuscript and provided funding, grant support and overight.

## Pre-publication history

The pre-publication history for this paper can be accessed here:

http://www.biomedcentral.com/1471-2407/9/356/prepub
